# Id-1 and Id-2 are markers for metastasis and prognosis in oesophageal squamous cell carcinoma

**DOI:** 10.1038/sj.bjc.6604035

**Published:** 2007-11-13

**Authors:** H-F Yuen, Y-P Chan, K-K Chan, Y-Y Chu, M L-Y Wong, S Y-K Law, G Srivastava, Y-C Wong, X Wang, K-W Chan

**Affiliations:** 1 Department of Pathology, Faculty of Medicine, The University of Hong Kong, Pokfulam, Hong Kong, China; 2 Department of Anatomy, Faculty of Medicine, The University of Hong Kong, Pokfulam, Hong Kong, China; 3 Department of Chemistry, Faculty of Science, The University of Hong Kong, Pokfulam, Hong Kong, China; 4 Department of Surgery, Faculty of Medicine, The University of Hong Kong, Pokfulam, Hong Kong, China

**Keywords:** Id proteins, ESCC, prognosis, metastasis

## Abstract

Id protein family consists of four members namely Id-1 to Id-4. Different from other basic helix–loop–helix transcription factors, they lack the DNA binding domain. Id proteins have been shown to be dysregulated in many different cancer types and their prognostic value has also been demonstrated. Recently, Id-1 has been shown to be upregulated in oesophageal squamous cell carcinoma (ESCC). However, the prognostic implications of Id proteins in ESCC have not been reported. We examined the expression of the Id proteins in ESCC cell lines and clinical ESCC specimens and found that Id protein expressions were dysregulated in both the ESCC cell lines and specimens. By correlating the expression levels of Id proteins and the clinicopathological data of our patient cohort, we found that M1 stage tumours had significantly higher nuclear Id-1 expression (*P*=0.012) while high nuclear Id-1 expression could predict development of distant metastasis within 1 year of oesophagectomy (*P*=0.005). In addition, high levels of Id-2 expression in both cytoplasmic and nuclear regions predicted longer patient survival (*P*=0.041). Multivariate analysis showed that high-level expression of Id-2 in both cytoplasmic and nuclear regions and lower level of nuclear Id-1 expression were independent favourable predictors of survival in our ESCC patients. Our results suggest that Id-1 may promote distant metastasis in ESCC, and both Id-1 and Id-2 may be used for prognostication for ESCC patients.

Oesophageal cancer is the eighth most common cancer and the sixth most common cause of cancer-related death worldwide with a 20-fold higher risk being observed in China when compared to other low-risk areas (Parkin *et al*, 2005). While the incidence of oesophageal adenocarcinoma in western countries is increasing, the predominant type of oesophageal cancer is still squamous cell carcinoma (Lam, 2000).

Id protein family is a group of helix–loop–helix transcription factors that have been implicated in different steps in carcinogenesis such as tumorigenesis, differentiation and metastasis (Benezra *et al*, 2001; Zebedee and Hara, 2001; Hasskarl and Munger, 2002; Sikder *et al*, 2003; Fong *et al*, 2004; Wong *et al*, 2004). Recent studies exploring the potential use of Id proteins as tumour markers have revealed that they play different roles in different types of cancer such that they might act as markers for progression, metastasis and prognosis in some cancer types. For instance, a high-level expression of Id-1 has been shown to be associated with poor prognosis of early-stage cervical cancer (Schindl *et al*, 2001), node-negative breast cancer (Schoppmann *et al*, 2003) and malignant melanoma (Straume and Akslen, 2005). Id-1 has also been shown to promote metastasis through increasing invasiveness and angiogenesis in breast and prostate cancer (Fong *et al*, 2003; Ling *et al*, 2005). High level of Id-2 has been demonstrated to reduce breast cancer cells invasiveness and therefore could act as a favourable prognostic marker for breast cancer patients (Stighall *et al*, 2005). In addition, the methylation status of Id-4 has been shown to correlate with poor prognosis in colorectal cancer (Umetani *et al*, 2004). Taken together, these results suggest that Id proteins are important in cancer initiation and progression, and that they may be useful prognostic markers.

Id-1 has been shown to be overexpressed in oesophageal squamous cell carcinoma (ESCC) (Hu *et al*, 2001). A recent study on functional roles of Id-1 in ESCC revealed that expression of Id-1 increased proliferation and decreased TNF-*α*-induced apoptosis in ESCC cell lines (Hui *et al*, 2006). These results suggest that Id-1 plays an important role in tumorigenesis of ESCC. However, the prognostic significance of Id-1 has not been adequately studied, and the expressions of other members of Id protein family in ESCC remain to be elucidated.

## MATERIALS AND METHODS

### Patients and specimens

The patient cohort consists of 84 ESCC Chinese patients who had undergone total oesophagectomy for ESCC in Queen Mary Hospital from 1998 to 2005. The clinical data collected prospectively are summarised in Table 1. Briefly, the patients recruited to this study had not received any chemoradiotherapy directed against ESCC prior to oesophagectomy. The oesophagectomy specimens were processed for routine histopathologic diagnosis. Tissue blocks were fixed in 10% buffered formalin overnight and then processed for paraffin embedding. Non-neoplastic oesophageal epithelium was selected from the upper resection margin of the oesophagectomy specimens. Clinical stage at the time of operation was classified according to the TNM staging system, while tumour grades were classified based on the World Health Organization classification (Hamilton and Aaltonen, 2000). Patients who developed distant metastasis within 1 year of oesophagectomy were regarded to have ESCC of high metastatic potential, while those who did not were considered to have ESCC of low metastatic potential. The median follow-up of our patient cohort is 12.6 months (ranged from 0.7 to 65.1 months).

### Cell lines

Immortalise oesophageal epithelial cell line NE1 was maintained in KSFM (Invitrogen, Carlsbad, CA, USA). ESCC cell lines EC18, EC109 and KYSE70 were maintained in RPMI-1640 (Invitrogen) with 10% FBS. HKESC2 was maintained in MEM (Invitrogen) with 20% FBS. KYSE30 and KYSE150 were maintained in 1 : 1 RPMI-1640/Ham's F12 medium with 10 and 2% FBS, respectively.

### Western blotting

Western blotting was performed as previously described (Kwok *et al*, 2005) using primary antibodies against Id-1 and Id-2 (Santa Cruz Biotechnology, Santa Cruz, CA, USA) at a concentration of 1 : 500 and 1 : 200, respectively. Actin was used as a loading control by using anti-actin antibody (Sigma, St Louis, MO, USA).

### Tissue microarray construction

Tissue microarray (TMA) was constructed with a Beecher Instruments tissue microarraryer (Beecher Instruments, Silver Spring, MD, USA). Three representative areas of each specimen, including 33 non-neoplastic oesophageal epithelium and 84 ESCC specimens, were selected by a pathologist (K-W Chan) and transferred to a TMA block using a 0.6 mm core diameter needle. Haematoxylin- and eosin-stained sections from each TMA block were checked by K-W Chan to ensure that adequate targeted areas had been included. The sample numbers varied slightly among staining results due to a variation in the number of interpretable specimens on TMA sections.

### Immunohistochemical staining

Immunohistochemical staining using Dako EnVision+ system-HRP (Dako Corporation, Glostrup, Denmark) was carried out according to the manufacturer's instructions. Polyclonal anti-Id-1 (C-20), Id-2 (C-20), Id-3 (C-20) and Id-4 (H-70) antibodies (Santa Cruz Biotechnology, Santa Cruz, CA, USA) were applied at a 1 : 600, 1 : 800, 1 : 200 and 1 : 50 dilution, respectively, for overnight incubation at 4°C. For peptide-blocking analysis used to verify antibody specificity, five-fold (w/w) excess of blocking peptide (Santa Cruz Biotechnology, Santa Cruz, CA, USA) was preincubated with the corresponding primary antibody overnight at 4°C before applying them to the TMA slides.

### Evaluation of immunohistochemical staining results

The stained sections were reviewed by two independent observers (K-W Chan and Y-P Chan) who had no prior knowledge of the clincopathological data of the patient cohort. The ‘hot spot’ method was employed in evaluation of the staining results. All three cores of each specimen in the TMA were individually scored, and the highest score was selected for later statistical analysis. The extent and intensity of cytoplasmic staining was graded by an arbitrary scale ranged from 0 to 3, representing negative (0), weak (1), moderate (2) and strong (3) staining, respectively. Negative and weak cytoplasmic staining was classified as low expression, while moderate and strong cytoplasmic staining was classified as high expression. Nuclear staining was scored by the percentage of nuclei positively stained in the whole core with at least 200 nuclei counted. Specimens with nuclear staining score lower than the mean percentage were classified as low expression and vice versa. An overall grade of expression was then assigned by combining both cytoplasmic and nuclear staining results such that only specimens having both high cytoplasmic and nuclear staining scores were graded as high overall staining. Staining patterns other than that were classified as low overall expression.

### Statistical analysis

Statistical analysis was performed using SPSS 14.0 software. Differences in expression level among different clinicopathological stages were analysed by *χ*^2^ test, Mann–Whitney *U*-test or Fisher's exact test where applicable. The association between expression level and the risk to develop distant metastasis within 1 year of oesophagectomy was estimated by odds ratio and 95% confidence intervals using binary logistic regression analysis. Survival curves between low- and high-level expressions of the four Id proteins were estimated by Kaplan–Meier analysis and compared by log-rank test. Cox regression analysis was performed to identify independent factors that predict patient survival. All factors with *P*-value <0.10 in the univariate analysis were allowed entry to the multivariate analysis. Backward stepwise selection was used with removal limits of *P*-value <0.05. A *P*-value <0.05 was considered significant in all the statistical analyses.

## RESULTS

### Expression of Id-1 and Id-2 in oesophageal cell lines

**Figure 1:**
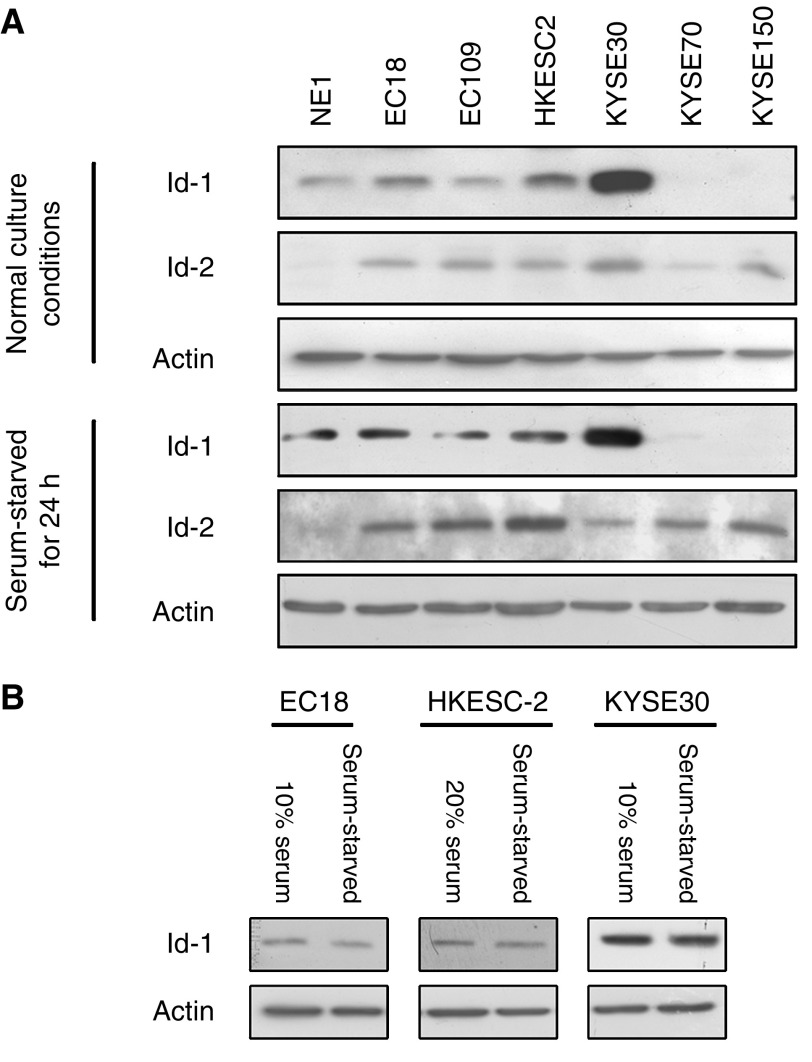
Western blot analysis of Id-1 and Id-2 expression in immortalized oesophageal epithelial and ESCC cells. Cancer cells (2 × 10^5^) were seeded in six-well plate with supplement of serum, according to the recommendation from the suppliers of the cell lines, as described in Materials and Methods. Medium was changed after 1 day with either serum-free medium or fresh serum-supplemented medium NE1 was cultured in KSFM with growth factors supplementation, and fresh medium was replaced after 1 day. Cells were harvested after 24 h. Protein was extracted and the expression level of Id-1 and Id-2 was then compared by Western blot analysis. Note that the expression of Id-1 was detected in four out of six ESCC cell lines while Id-2 was expressed in all ESCC cell lines tested. (**B**) The expression level of Id-1 in three ESCC cancer cell lines with or without serum supplementation. Id-1 expression level was slightly reduced when the cells were serum-starved for 24 h.

As shown in Figure 1, Id-1 was detected in four out of six ESCC cell lines with a high expression in three cell lines investigated. Id-2 was expressed in all six ESCC cell lines and was barely detected in NE1. These results show that Id-1 and Id-2 are dysregulated in ESCC as compared to immortalised oesophageal cell line NE1. In contrast, we found that Id-1 expression was slightly reduced when the cells were serum-starved. This result suggests that Id-1 expression in ESCC is only slightly affected by the presence or absence of serum.

### Id protein expressions in ESCC and non-neoplastic oesophageal epithelium specimens

**Figure 2:**
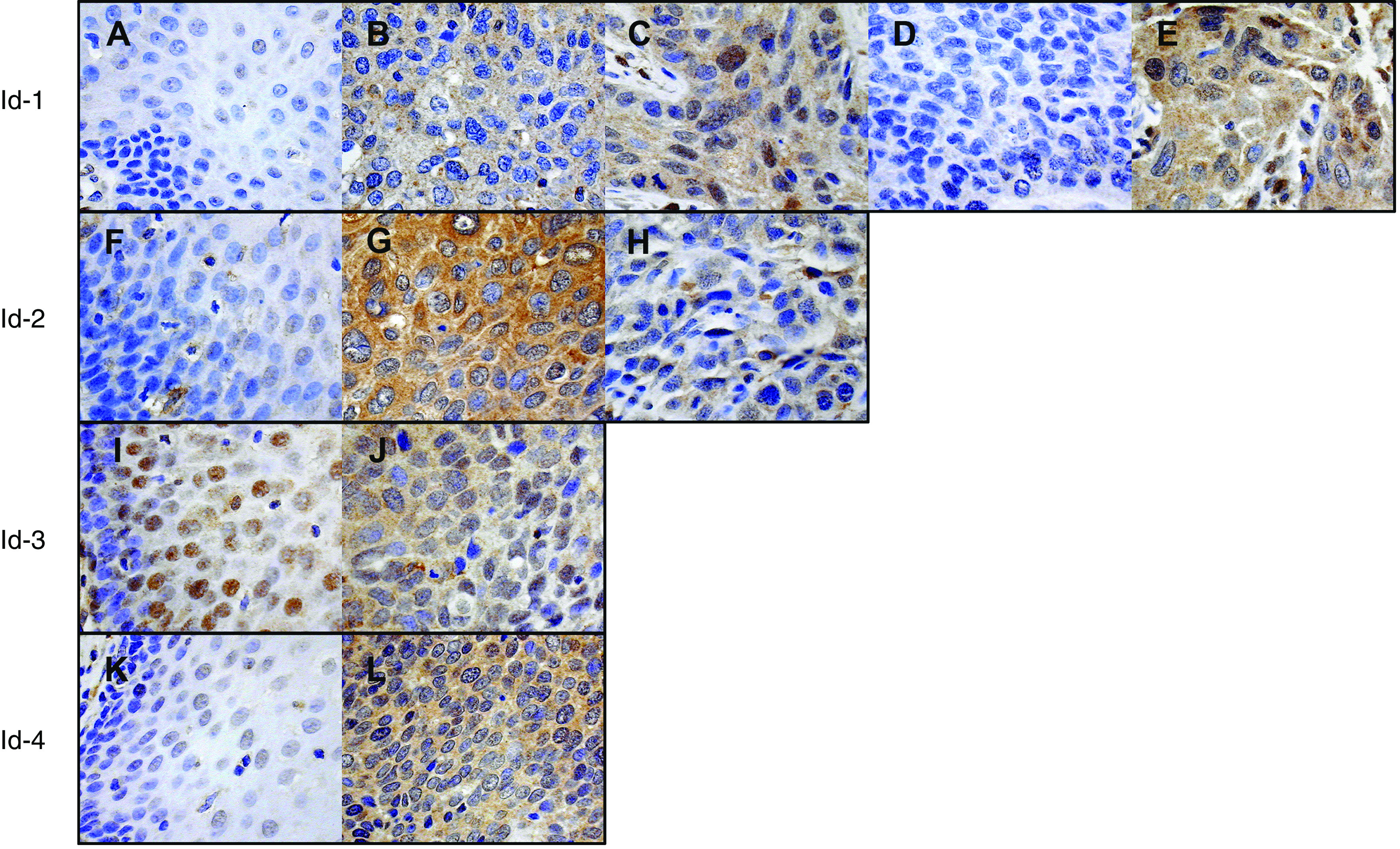
Representative results of immunohistochemistry of Id-1 to Id-4 expression in oesophageal specimens. (Panel 1) Id-1 expression was negative to weak in non-neoplastic oesophageal epithelium (**A**), but increased in ESCC specimens (**B**–**E**). While a low level of nuclear Id-1 staining was detected in primary ESCC at M0 stage (**B**), a significantly higher level of nuclear Id-1 staining was observed in tumours at M1 stage (**C**). ESCC at T2 stage (**D**) had weak cytoplasmic Id-1 staining while a high-level cytoplasmic Id-1 expression was detected in T4 stage tumour (**E**). (Panel 2) Id-2 staining was relatively weak in normal oesophageal epithelium (**F**) while was higher in tumour specimens (**G** and **H**). High level of cytoplasmic Id-2 staining was observed in well-differentiated tumours (**G**) but was significantly lower in poorly differentiated tumours (**H**). (Panel 3) Id-3 staining was high in both normal oesophageal epithelium (**I**) and ESCC specimens (**J**), with mainly nuclear staining in the former one and both cytoplasmic and nuclear staining in the later one. (Panel 4) Id-4 expression was relatively weak in normal oesophageal epithelium (**K**) and higher in tumour specimens (**L**). Image was captured at × 400 magnification.

As shown in Figure 2, Id protein expressions were dysregulated in human ESCC specimens when compared to their non-neoplastic counterparts. Non-neoplastic oesophageal epithelium expressed a lower level of Id-1 than the tumour specimens. A majority (85%, 28 out of 33) of non-neoplastic oesophageal epithelium specimens showed negative cytoplasmic and nuclear Id-1 staining, while 36% (29 out of 80) and 29% (23 out of 80) of ESCC specimens showed moderate to strong cytoplasmic Id-1 staining and positive nuclear Id-1 staining, respectively. Both nuclear and cytoplasmic Id-2 expressions were relatively low in non-neoplastic oesophageal epithelium with 53% (17 out of 32) of specimens showed negative to weak staining. However, as many as 87% (69 out of 79) of ESCC specimens showed moderate to strong cytoplasmic Id-2 staining and 66% (52 out of 79) showed positive nuclear Id-2 staining. These results suggest that Id-1 and Id-2 were differentially expressed in non-neoplastic oesophageal epithelium and ESCC specimens. In addition, overexpression of Id-1 and Id-2 might be important in neoplastic transformation of ESCC.

In contrast, expressions of Id-3 and Id-4 did not differ much between non-neoplastic epithelium and tumour tissues. Id-3 was positively detected in 94% (31 out of 33) of non-neoplastic specimens and in 90% (72 out of 80) of ESCC specimens. While 17 out of 33 (52%) non-neoplastic oesophageal epithelium samples expressed a low level of Id-4, 36 out of 80 (45%) ESCC specimens showed similar low expression. Id-3 was mainly expressed in the nucleus and sometimes in cytoplasm of non-neoplastic oesophageal epithelium, but its expression in ESCC was generally both cytoplasmic and nuclear. Id-4 was mainly detected in the nucleus of non-neoplastic oesophageal epithelium samples, while its expression in cytoplasm is more frequent in ESCC.

### Id protein expressions correlated with clinicopathological parameters

**Figure 3:**
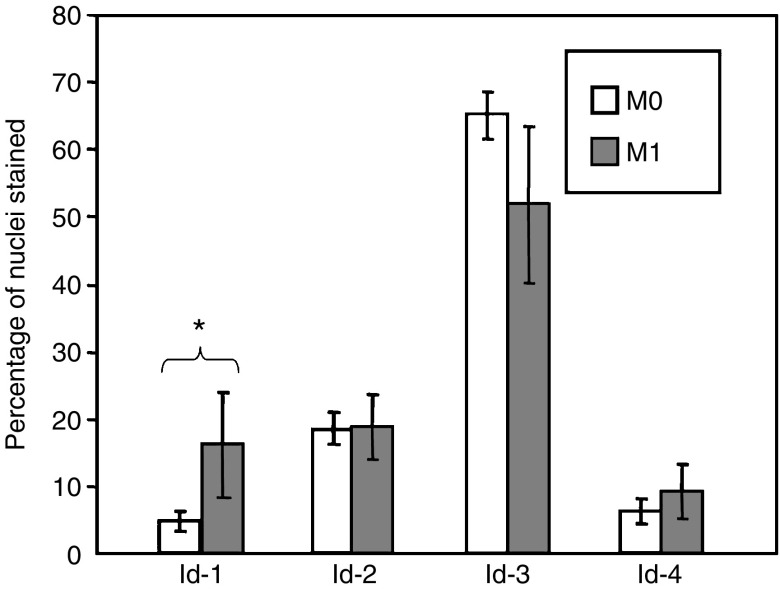
Nuclear expression of Id proteins in ESCC of different M stages. Significantly higher nuclear Id-1 expression was detected in M1 stage tumours when compared to M0 stage tumours (*P*=0.012). No significant association was found between nuclear Id-2, -3 and -4 expression and M stage.

As shown in Figures 2 and 3, we found that primary ESCC at M1 stage had significantly higher (*P*=0.012) nuclear expression of Id-1 (16.13±7.82%) than those at M0 stage (4.76±1.55%). Binary logistic regression was then employed to estimate the odds ratio for patients having high-level nuclear expression of Id-1 in their primary ESCC to develop distant metastasis within 1 year. Primary ESCC tumours with more than 11.86% (mean percentage of nuclei stained positive in ESCC specimens) of nuclei stained positive for Id-1 were regarded as having high and others as having low nuclear expression. The results showed that patients with high-level nuclear expression of Id-1 in their primary ESCC were at a higher risk to develop distant metastasis within 1 year (odds ratio: 4.615, 95% confidence interval (CI): 1.596−13.348, *P*=0.005).

**Figure 4:**
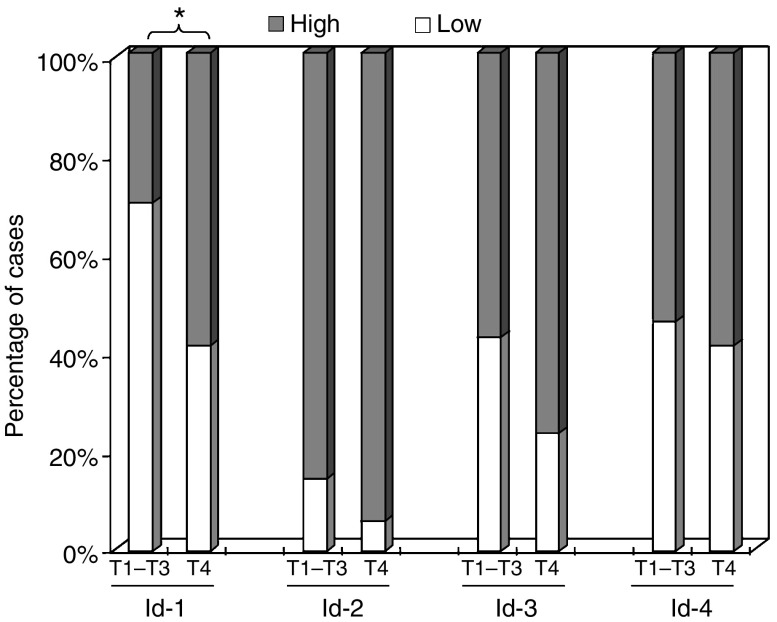
Cytoplasmic expression of Id proteins in ESCC of different T stages. Significantly higher percentage of patients with T4 stage tumour had a higher cytoplasmic Id-1 expression when compared to patients with T1–T3 stages tumour (*P*=0.045). No significant association was found between cytoplasmic Id-2, -3 and -4 expressions and T stage.

In contrast, we observed that 59% of primary ESCC at the T4 stage showed a high cytoplasmic Id-1 expression, whereas only 30% of ESCC at the lower T stages with similar staining extent (Figure 4). By statistical analysis, we demonstrated that high-level cytoplasmic Id-1 expression was significantly (*P*=0.045) associated with T4 tumour.

**Figure 5:**
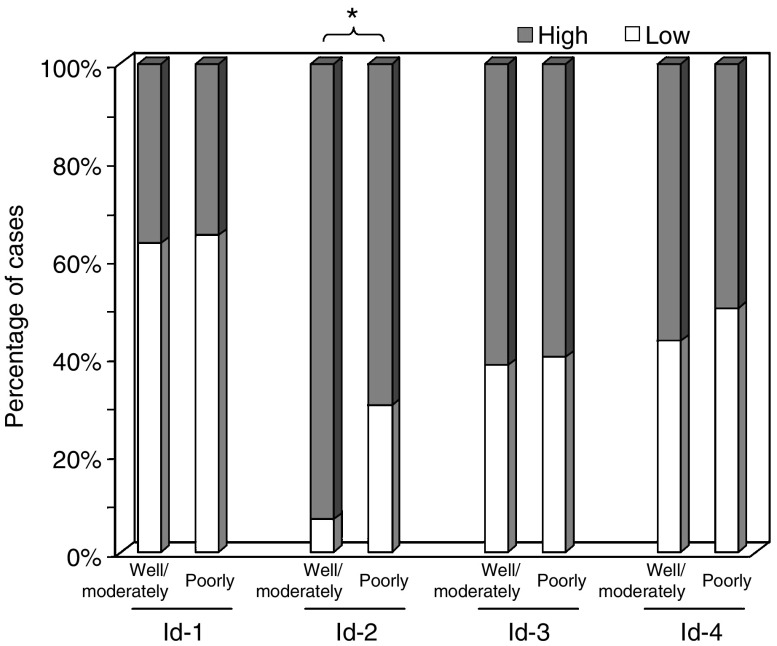
Cytoplasmic expression of Id proteins in ESCC of different histological grades. Significantly higher percentage of patients with poorly differentiated tumour had a low cytoplasmic Id-2 expression when compared to patients with well or moderately differentiated tumour (*P*=0.013). No significant association was found between cytoplasmic Id-1, -3 and -4 expression and histological grade.

In addition, a statistically significant association was observed between low-level cytoplasmic Id-2 expression and poor differentiation of the tumour (*P*=0.013). While 30% of poorly differentiated ESCC expressed low-level cytoplasmic Id-2, the same was observed in only 7% of well or moderately differentiated tumours (Figure 5). However, expression levels of both Id-3 and Id-4 were not significantly associated with any of the clincopathological parameters analysed. These results suggest that dysregulations of Id-1 and Id-2 are involved in tumour progression and metastasis of ESCC.

**Figure 6:**
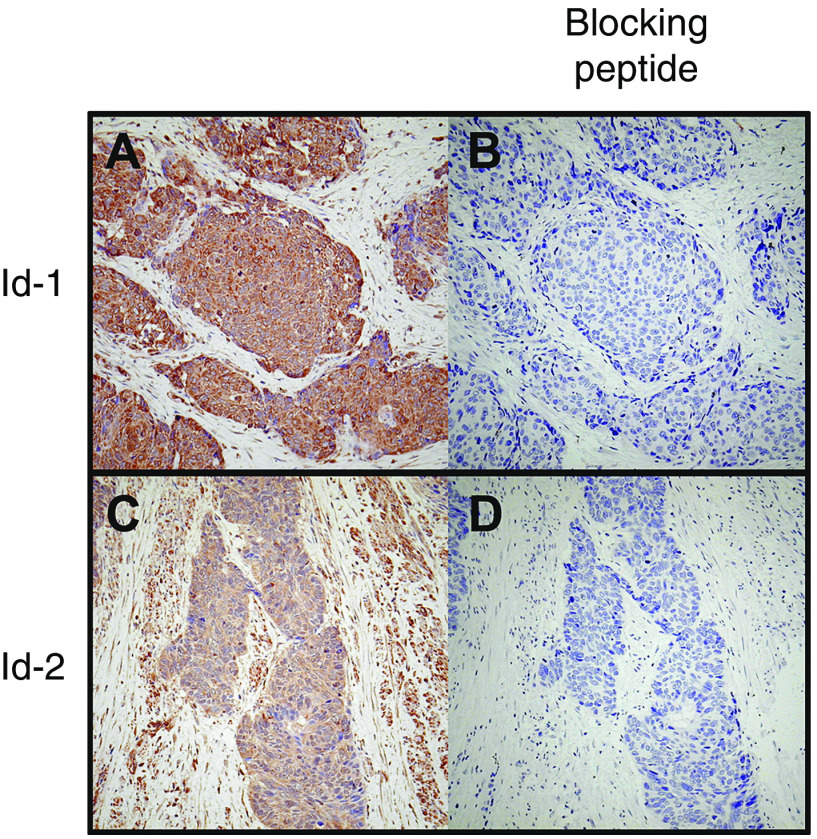
Peptide neutralization of Id-1 and Id-2. (Panel 1) Id-1 staining was strong in a particular ESCC case (**A**) but was negative when Id-1 blocking peptide was added (**B**). (Panel 2) Staining of Id-2 was strong in a particular ESCC case (**C**) but was negative when Id-2 blocking peptide was added (**D**).

To investigate the specificity of Id-1 and Id-2 antibodies in immunohistochemistry, we performed a peptide neutralisation analysis. By preincubating the primary antibody with five-fold (w/w) excess of corresponding blocking peptide, the signal was almost completely abolished (Figure 6). These results confirmed the specificity of the antibodies used.

### Id-1 and Id-2 are independent prognostic factors for survival in our ESCC cohorts

**Figure 7:**
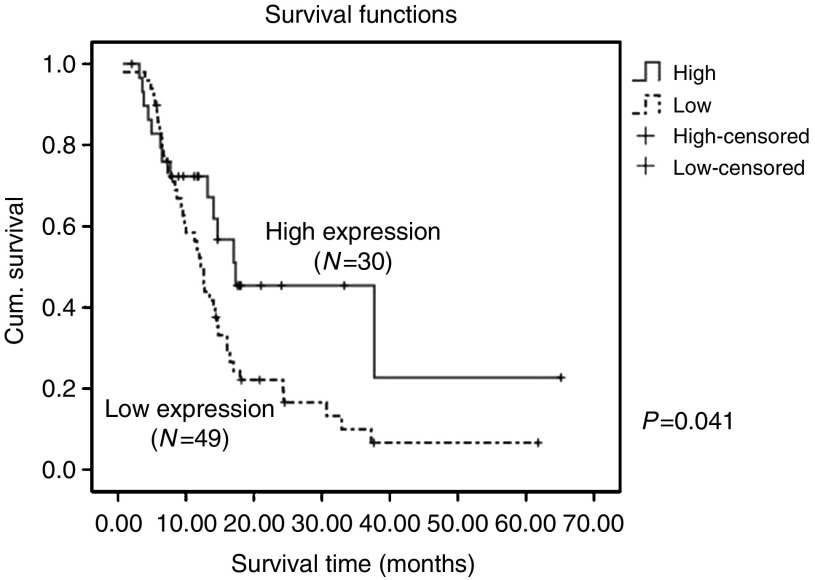
Kaplan–Meier analysis of Id-2 expression and overall patient survival. High level of both cytoplasmic and nuclear expression of Id-2 significantly (*P*=0.041) predicted better overall patient survival.

By Kaplan–Meier analysis, a high overall grade combining cytoplasmic and nuclear expression of Id-2 significantly (*P*=0.041) predicted better survival in our cohort (Figure 7). The mean survival was 28.9 (95% CI: 16.7−41.1) months for patients with high overall Id-2 expression and 16.8 (95% CI: 12.3−21.1) months for those with low overall Id-2 expression. To identify independent predictors for survival, univariate and multivariate Cox-regression analyses were performed. All the clinicopathological parameters and Id protein expression levels including age, sex, TNM stage, histological stage, and cytoplasmic, nuclear and overall Id protein expressions were entered into univariate Cox-regression analysis. We found that only T4 stage (*P*=0.007), M1 stage (*P*=0.026) and low overall Id-2 expression (*P*=0.045) were significant predictors of poor survival in univariate analysis (Table 2). Factors with *P*⩽0.10 in univariate analysis were allowed entry to multivariate Cox-regression analysis. We found that increasing age (*P*=0.017), male sex (*P*=0.019), increasing nuclear Id-1 expression (*P*=0.011) and low overall Id-2 expression (*P*=0.020) individually contributed significantly to poor prognosis as shown by the multivariate analysis (Table 2).

## DISCUSSION

Id proteins have been recently demonstrated to promote cancer progression and metastasis and are potential prognostic factors in different types of cancer. In this study, we aimed to investigate how this knowledge could be applied to further our understanding of the pathogenesis and progression of ESCC. The expression levels of Id proteins in ESCC and non-neoplastic oesophageal epithelium in corresponding oesophagectomy specimens were studied by immunohistochemistry and the results were correlated with clinicopathological parameters. We observed that primary tumours at M1 stage had a significantly higher nuclear Id-1 expression than M0 stage tumours (*P*=0.012). A high level of cytoplasmic Id-1 expression was significantly associated with T4 stage tumour (*P*=0.045). In contrast, low-level cytoplasmic Id-2 expression was significantly associated with poorly differentiated tumours (*P*=0.013). In addition, high level of overall Id-2 expression in primary tumour predicted better survival of ESCC patient in Kaplan–Meier analysis (*P*=0.041). By multivariate Cox-regression analysis, we were able to demonstrate that increasing nuclear Id-1 expression (*P*=0.011) and low overall Id-2 expression (*P*=0.020) were independent predictors of poor overall survival in our ESCC patients.

Id-1 has been shown to promote metastasis in several different types of cancer. Overexpression of Id-1 promotes mammary epithelial cell invasion (Desprez *et al*, 1998), and reduced expression of Id-1 in metastatic breast cancer cells leads to a decrease in invasiveness *in vitro* (Fong *et al*, 2003). Overexpression of Id-1 in prostate cancer cells also promotes metastasis through increased angiogenesis (Ling *et al*, 2005) and invasiveness (Coppe *et al*, 2004). Reduced expressions of Id-1 and Id-3 have been demonstrated to inhibit the metastatic ability of gastric cancer cells (Tsuchiya *et al*, 2005), and Id-1 expression has been shown to be associated with the presence of invasion in endometrial carcinoma (Takai *et al*, 2001) and tumour angiogenesis in human pancreatic cancer (Lee *et al*, 2004). In lines with these results, we found that primary ESCC at M1 stage had a significantly (*P*=0.012) higher nuclear expression of Id-1 than the M0 stage tumours. In addition, by binary logistic regression, we observed that a high nuclear expression of Id-1 in primary ESCC tumours significantly predicted the development of distant metastasis of the corresponding patients within 1 year of oesophagectomy (odds ratio: 4.615, 95% CI: 1.596–13.348, *P*=0.005). In addition, a high level of cytoplasmic Id-1 expression was significantly (*P*=0.045) correlated with T4 stage tumours. These results suggest that upregulation of Id-1 in ESCC might promote distant metastasis and cancer progression. *In vitro* experiments are being undertaken to investigate the functional role of Id-1 in metastatic progression of ESCC.

Id-2 has been suggested to promote cellular differentiation in normal murine mammary epithelial cells (Parrinello *et al*, 2001). In breast cancer, Id-2 expressed at high levels maintains the cancer cells in a non-aggressive phenotype (Itahana *et al*, 2003). In addition, high level of Id-2 expression has been shown to reduce the invasiveness of breast cancer cells and can act clinically as a favourable prognostic marker (Stighall *et al*, 2005). Similar results have been obtained in intestinal epithelium where Id-2 promotes differentiation and inhibit tumorigenesis (Russell *et al*, 2004). Our results on Id-2 expression in ESCC also showed that low level of cytoplasmic Id-2 expression was significantly (*P*=0.013) associated with poor tumour differentiation. Moreover, high level of both nuclear and cytoplasmic expression of Id-2 provides favourable prognosis for our ESCC patients, with mean survival of 28.9 (95% CI: 16.7–41.1) months when compared to those having low overall Id-2 expression (16.8 (95% CI: 12.3–21.1) months).

In the present study, we observed that Id-2 expression was low in the immortalised oesophageal cell line NE1 and the non-neoplastic oesophageal epithelium samples showed lower Id-2 expression than their cancer counterparts. These results suggested that Id-2 promotes neoplastic transformation of oesophageal epithelial cells. Id-2 has been shown to bind and inhibit the function of retinoblastoma protein, a tumour suppressor protein (Iavarone *et al*, 1994; Lasorella *et al*, 1996) and therefore has been implicated in tumour progression in several cancer types (Maruyama *et al*, 1999; Fukuma *et al*, 2003; Coppe *et al*, 2004). We speculated that overexpression of Id-2 might promote neoplastic transformation of oesophageal epithelium through destruction of the normal cell cycle checkpoint and increased proliferation. After onset of other oncogenic events, reduced Id-2 expression might increase aggressiveness of ESCC cancer cells through dedifferentiation and leads to poor patient survival.

To test whether Id-1 and Id-2 might act as independent prognostic factors in our patient cohort, Cox-regression with backward stepwise selection was carried out. The results (Table 2) showed that higher nuclear expression of Id-1 (*P*=0.011) and low-level overall expressions of Id-2 (*P*=0.020) were independent predictors for poor prognosis in our cohort of ESCC patients.

In summary, our results suggest that overexpression of Id-1 can promote neoplastic transformation of normal oesophageal epithelium, ESCC progression and the development of distant metastasis. In contrast, expression of Id-2 might inhibit the emergence of a more aggressive phenotype of ESCC. We provide new evidence that Id-1 and Id-2 may be useful prognostic indicators of metastatic potential of ESCC and survival of patients, respectively.

## Figures and Tables

**Table 1:** Patients' clinical and pathologic features

	**Number of cases**	**%**	**Median (range)**
Age (years)	84		67 (22–87)
*Sex*			
Male	65	77	
Female	19	23	
			
*T stage*			
T1	2	2	
T2	14	17	
T3	51	61	
T4	17	20	
			
*N stage*			
N0	31	37	
N1	53	63	
			
*M stage*			
M0	69	82	
M1	15	18	
			
*Histological grade*			
Well differentiated	13	16	
Moderately differentiated	48	57	
Poorly differentiated	23	27	
			
*Metastatic status*	64		
Non-metastatic	36	56	
Metastatic (within 1 year)	28	44	

**Table 2:** Cox regression analyses of overall survival

	**Univariate analysis**		**Multivariate analysis**	
**Clinicopathological parameters**	**RR (95% CI)**	***P*-value**	**RR (95% CI)**	***P*-value**
Age (*n*=84)	1.024 (1.000–1.049)	0.055	1.033 (1.006–1.060)	0.017
				
*Sex*				
Female (*n*=19)	1	Reference	1	Reference
Male (*n*=65)	1.983 (0.993–3.959)	0.052	2.418 (1.158–5.047)	0.019
				
*T stage*				
T1/T2 (*n*=16)	1	Reference		
T3 (*n*=51)	1.339 (0.639–2.803)	0.439		
T4 (*n*=17)	3.191 (1.380–7.377)	0.007		
				
*M stage*				
M0 (*n*=69)	1	Reference		
M1 (*n*=15)	2.077 (1.089–3.960)	0.026		
				
Nuclear Id-1 expression (*n*=79)	3.610 (0.918–14.197)	0.066	7.361 (1.588–34.109)	0.011
				
*Overall Id-2 expression*				
Low (*n*=49)	1.862 (1.014–3.421)	0.045	2.151 (1.127–4.107)	0.020
High (*n*=30)	1	Reference	1	Reference

CI=confidence interval; RR=relative risk.
